# Optimization and analysis of a quantitative real-time PCR-based technique to determine microRNA expression in formalin-fixed paraffin-embedded samples

**DOI:** 10.1186/1472-6750-10-47

**Published:** 2010-06-23

**Authors:** Rashmi S Goswami, Levi Waldron, Jerry Machado, Nilva K Cervigne, Wei Xu, Patricia P Reis, Denis J Bailey, Igor Jurisica, Michael R Crump, Suzanne Kamel-Reid

**Affiliations:** 1 Division of Applied Molecular Oncology, Ontario Cancer Institute, University Health Network, Toronto, ON, Canada; 2 Department of Laboratory Medicine and Pathobiology, University of Toronto, Toronto, ON, Canada; 3 Division of Signaling Biology, Ontario Cancer Institute; 4 Campbell Family Institute for Cancer Research, University Health Network, Toronto, ON, Canada; 5 Department of Biostatistics, Princess Margaret Hospital, University Health Network, Toronto, ON, Canada; 6 Department of Pathology, Toronto General Hospital, University Health Network, Toronto, ON, Canada; 7 Department of Computer Science, University of Toronto, Toronto, ON, Canada; 8 Department of Medical Biophysics, University of Toronto, Toronto, ON, Canada; 9 Division of Medical Oncology, Princess Margaret Hospital, University Health Network, Toronto, ON, Canada

## Abstract

**Background:**

MicroRNAs (miRs) are non-coding RNA molecules involved in post-transcriptional regulation, with diverse functions in tissue development, differentiation, cell proliferation and apoptosis. miRs may be less prone to degradation during formalin fixation, facilitating miR expression studies in formalin-fixed paraffin-embedded (FFPE) tissue.

**Results:**

Our study demonstrates that the TaqMan Human MicroRNA Array v1.0 (Early Access) platform is suitable for miR expression analysis in FFPE tissue with a high reproducibility (correlation coefficients of 0.95 between duplicates, p < 0.00001) and outlines the optimal performance conditions of this platform using clinical FFPE samples. We also outline a method of data analysis looking at differences in miR abundance between FFPE and fresh-frozen samples. By dividing the profiled miR into abundance strata of high (Ct<30), medium (30≤Ct≤35), and low (Ct>35), we show that reproducibility between technical replicates, equivalent dilutions, and FFPE *vs*. frozen samples is best in the high abundance stratum. We also demonstrate that the miR expression profiles of FFPE samples are comparable to those of fresh-frozen samples, with a correlation of up to 0.87 (p < 0.001), when examining all miRs, regardless of RNA extraction method used. Examining correlation coefficients between FFPE and fresh-frozen samples in terms of miR abundance reveals correlation coefficients of up to 0.32 (low abundance), 0.70 (medium abundance) and up to 0.97 (high abundance).

**Conclusion:**

Our study thus demonstrates the utility, reproducibility, and optimization steps needed in miR expression studies using FFPE samples on a high-throughput quantitative PCR-based miR platform, opening up a realm of research possibilities for retrospective studies.

## Background

MicroRNAs (miRs) are small, non-coding RNA molecules of 17-27 nucleotides in length, involved in gene regulation at the post-transcriptional level [1]. They inhibit translation by partially or totally binding to the complementary 3' UTR of their target mRNAs within the multiprotein RNA-induced silencing complex (RISC). Full complementarity between a miR and its target mRNA results in mRNA degradation; partial complementarity leads to inhibition of mRNA translation. The literature on miRs has grown exponentially within the past decade as these small molecules have demonstrated various roles in early development, cell proliferation, differentiation, apoptosis and oncogenesis [1-6]. Therefore, techniques to analyze and characterize their expression are a key to understanding their role in disease and development.

Anatomical pathology laboratories worldwide contain a vast stock of samples that can potentially be used for analysis of disease states. These are in the form of formalin-fixed, paraffin-embedded (FFPE) samples that are stored for up to 20 years and possibly longer depending on professional or governmental guidelines. Given the length of the storage period for these samples, extensive retrospective analyses with significant periods of clinicopathological follow-up for patient studies can be carried out.

Embedding of samples in paraffin after formalin fixation is a standard of practice [7]. This poses a problem for gene expression studies, because formalin fixation and the subsequent ethanol processing results in the formation of cross-links between RNA molecules and proteins, leading to a significant reduction in recovery of RNA from FFPE tissue. Formalin fixation and ethanol processing also leads to the production of mono-methylol and ethoxylated adducts with the bases of nucleic acids, as well as depurination fragments [8-10], reducing the efficiency of reverse transcription and negatively affecting downstream applications [7].

Despite these challenges, extraction of miRs from FFPE tissue is possible, as the small size contributes to their stability during fixation and processing [11]. miRs may also be protein protected by the RISC complex and therefore less susceptible to RNA degradation in comparison to mRNAs [12], however they do not totally escape degradation even in fresh-frozen tissues [13]. Despite this, miRs are more stable and more easily recovered from FFPE tissue than mRNAs [7], and may be a better choice for expression profiling when using FFPE samples [7,11]. Previously it has been shown that regardless of fixation time or age of tissue blocks, quantitative real-time PCR data for two miRs (miR-16 and miR-122) can be generated from FFPE tissues from different sites [14]. Although other studies have shown miR expression analysis using FFPE samples [11,12,14-20], to date, only one other study has shown the utility of the TaqMan Low Density Array technology for high-throughput miR expression profiling in archival and paired fresh-frozen tissue [21]. Here we demonstrate the use of quantitative real-time PCR (qRT-PCR) in high-throughput analysis of miR expression for 365 miRs using the TaqMan Low Density Array technology in clinical FFPE samples. We examine the utility of the TaqMan Human MicroRNA Array v1.0 (Early Access) microfluidics platform (Applied Biosystems) in the study of miR expression, and demonstrate the optimization steps needed to carry out an experiment using this platform. Comparison of high-throughput analysis of miR expression using FFPE samples and paired fresh-frozen samples is also shown. This may be of use to many researchers, due to the availability of archival samples in pathology laboratories worldwide.

## Results

### Optimizing input RNA concentration for TaqMan Human MicroRNA Array

RNA from a FFPE benign, reactive lymph node was reverse transcribed and the resultant cDNA applied to the TaqMan array. Varying RNA concentrations (10, 25, 50, 100 and 200 ng/μL) were used to assess the sensitivity of the TaqMan array platform to detect small quantities of RNA. All reverse transcription reaction products were diluted 15× prior to adding TaqMan 2× Universal PCR Master Mix, No AmpErase UNG and undergoing qRT-PCR. Duplicate plates were run to examine the reproducibility of the method. Ct values were determined for each individual miR for each sample. The Pearson correlation coefficients for each pair of samples were: 0.92 (10 and 25 ng/μL), 0.94 (50 and 100 ng/μL) and 0.95 (200 ng/μL). cDNA dilution factors in this analysis were kept constant at 15× for each plate.

Mixed model regression analysis was applied in order to adjust for repeated measures of the same sample and potential dilution effects. Using this analysis, the adjusted Pearson correlation coefficient was 0.95 (p < 0.00001). These results showed that RNA from FFPE tissues can be reliably amplified by qRT-PCR, and the quantification measures are reproducible between duplicates. The correlation coefficients obtained between duplicate pairs of input RNA amounts were high, ranging from 0.92 to 0.95, increasing with higher RNA concentrations.

**Figure 1:**
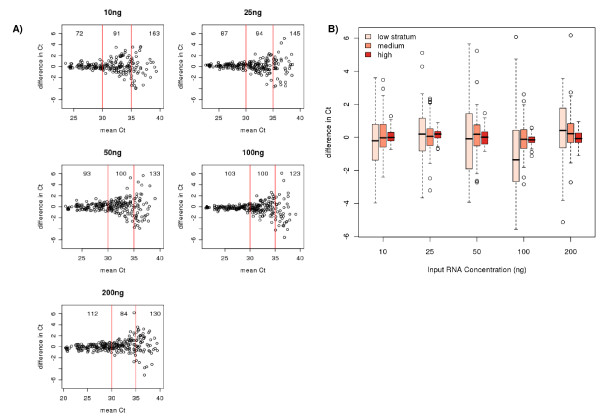
**Ct values according to different input RNA concentrations**. A) Plot of the difference in Ct values vs. mean Ct between duplicate plates for varying input RNA concentrations. Red lines indicate divisions between high (Ct<30), medium (30≤Ct≤35) and low (Ct>35) abundance strata. The total number of miRs is shown within each stratum for each RNA concentration (e.g., 72 miRs have Ct values <30 at the 10 ng RNA dilution compared to 112 miRs at the 200 ng dilution). B) Boxplots depicting the median of the absolute difference in Cts between duplicate plates for each miR abundance stratum according to input RNA concentration.

In qRT-PCR, accurate and reproducible data are dependent on the Ct value. Low Ct values indicate the presence of higher template abundance, and are usually associated with a higher reproducibility and lower variability [22]. We thus stratified our data based on Ct values and divided miRs into three strata of high (Ct values <30), medium (Ct values from 30-35) and low abundance (Ct values >35). To establish these cutoffs for miR abundance, we plotted the absolute value of the difference between duplicate Ct measurements as a function of the duplicate mean, for all input RNA concentrations. Measurements where one or both of the duplicate Ct values are exactly 40 are removed. Using the R statistics package (R Development Core Team, 2008), we applied a cubic spline function to fit a smooth curve to the data (Additional file 1, Figure S1). miRs with Ct values <30 showed the least variability. In contrast, miRs with Ct values >35 were highly variable. We examined how the various RNA concentrations affected miR abundance and Ct reproducibility in each stratum. As seen in Figure 1, the high abundance stratum is associated with consistently high reproducibility between biological and technical replicates. Reproducibility decreases across the medium stratum, and is consistently poor in the low stratum. Results from this analysis also demonstrated a shift in the number of miRs from the low and medium abundance strata to the high abundance stratum with increasing concentrations of input RNA (Figure 1A).

Boxplots outlining the summary statistics for the difference in Ct values between duplicate plates at each input RNA concentration were developed to determine which input RNA concentration has the least variation between duplicate plates (Figure 1B). From this analysis, the variability between duplicate plates decreases from the low to the medium and high abundance strata. In addition, this analysis reveals that an input RNA concentration should be maximized wherever possible to improve technical reproducibility.

### Assessing well failures at varying input RNA concentrations and cDNA dilutions

In order to assess the amplification efficiency when using this TaqMan array platform, we counted the number of wells that did not amplify in the plates (defined as well failures). Since the qRT-PCR reactions were set up to run for 40 cycles, a well was marked as a "failure" when, for the same miR, a Ct = 40 was recorded for one plate and a Ct < 40 was recorded for the duplicate. When both wells had a Ct = 40, Ct values for that miR were compared to the Ct values obtained at other concentrations, for the same sample, and also compared to the positive control, Universal RNA. If Ct values < 40 were obtained at other concentrations or on the plates containing Universal RNA for that miR, both wells were deemed to be "failures". The total number of well failures were assessed, for each plate, at each RNA concentration, and averaged among duplicate plates. The percentage of well failures was calculated as follows: Average percentage of well failures = Average well failure between duplicate plates/Number of miRs expressed*100 (Additional file 2, Figure S2). The percentage of well failures decreases with increasing input RNA concentration (Additional file 2, Figure S2). From these results, the lowest percent well failure (<5%) is seen with the highest input RNA concentration (200 ng/μL).

We also wanted to observe the effect of changing the cDNA dilution on well failures. For this experiment, input RNA concentrations were held constant at 200 ng/μL and 100 ng/μL. After reverse transcription at these input RNA concentrations, cDNA samples were diluted at 62.5× (as outlined by the manufacturer's protocol), 30×, and 15×, respectively. Well failures were assessed as previously outlined. Since we observed high correlation coefficients in the previous experiments, demonstrating robustness of the platform, only one plate per cDNA dilution was run to assess well failure. In this case, we define a well failure when a Ct value of 40 was obtained for any given miR known to be expressed in that same sample.

Our analysis showed that the percentage of well failures increases with cDNA dilution, and well failures are higher with lower input RNA concentration at the same cDNA dilution factor (Additional file 2, Figure S2). From these results, we conclude that the optimal input RNA concentration is 200 ng/μL, with a 15× cDNA dilution.

### Determining the optimal amount of RNA input concentration and cDNA dilution

Given the results above, we wanted to determine whether miR expression would be the same for equivalent input RNA concentrations and cDNA dilutions, defined here as equivalent samples. We tested the following RNA input and cDNA dilutions: 200 ng/μL at a 15× cDNA dilution, 100 ng/μL at a 7.5× cDNA dilution and 66.7 ng/μL at a 5× cDNA dilution. The Pearson correlation coefficient between these samples was > 0.944 (Range: 0.944-0.96) and the percentage of well failures (assessed as described earlier) between the samples was similar, ranging from 4-7% (Additional file 3, Table S1).

We also investigated the concordance in miR expression between equivalent samples. In this analysis, we compared the 200 ng/μL input RNA at 15× cDNA dilution with its duplicate and with the 100 ng/uL RNA at 7.5× cDNA dilution and 66.7 ng/uL RNA at 5× cDNA dilution. We stratified each miR by its mean Ct value across these conditions, and determined the correlation between these conditions in each stratum (low, medium and high abundance).

**Figure 2:**
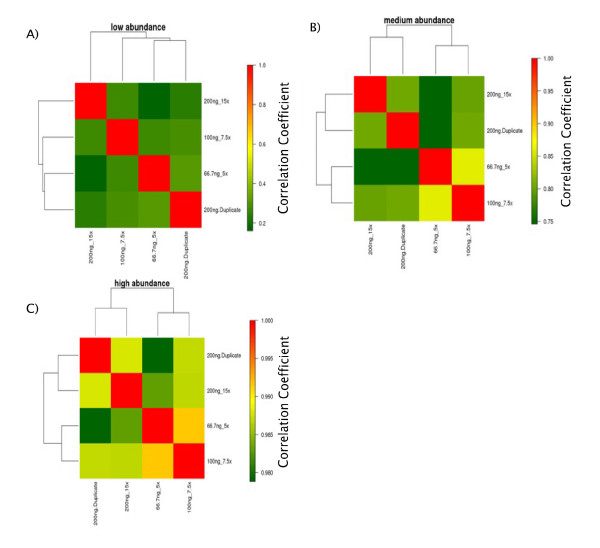
**Clustering heat-maps and pair-wise correlations for equivalent samples**. The pair-wise correlations between equivalent samples are shown within A) low abundance stratum: The low abundance miRs have a low correlation coefficient (range: 0.16-0.31) regardless of the concentration and dilution factor used. B) medium abundance stratum, showing higher correlation coefficients (range: 0.75-0.86) compared to the low abundance stratum. C) high abundance stratum: In this stratum we detect the highest correlation coefficients (range: 0.98-0.99) between samples. Note that the scales are different for each abundance stratum, reflecting the respective correlation coefficients.

The concordance rate was highly dependent on transcript abundance, as seen in the correlation heat-maps produced from these data (Figures 2A-C). We found a similar correlation between equivalent samples, with no statistically significant differences among the pair-wise correlations. As would be expected for equivalent samples, there were no significant differences in the distribution of miRs among the abundance strata by the chi-square test (χ^2 ^= *3.3, df = 6, p = 0.77*) (Additional file 4, Table S2).

### Comparison between fresh-frozen and FFPE clinical samples

**Figure 3:**
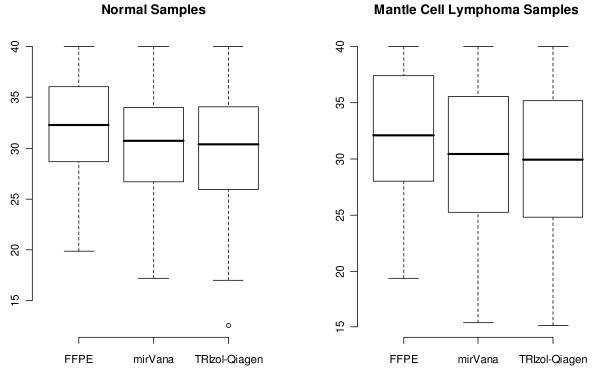
**Ct values according to different extraction methods for paired fresh-frozen and FFPE tissues**. Comparison of the summary statistics (mean Ct, standard deviation, and overall range of Ct values) using raw Ct scores obtained from the normal lymph nodes and mantle cell lymphoma samples extracted using three separate RNA extraction methods. On comparison of the FFPE summary statistics to those from the mirVana and TRIzol-Qiagen extraction protocols, we see that the mean Ct values obtained from the FFPE samples are significantly higher than those obtained from the mirVana and TRIzol-Qiagen extraction protocols (p < 0.0001 and p < 0.0001 respectively).

Pearson correlation coefficients were calculated for 3 tumour samples and 3 benign lymph nodes, according to the different extraction methods used. High correlation values of up to 0.87 (p < 0.0001; calculated based on an asymptotic t-distribution) are seen when comparing FFPE to fresh-frozen samples, regardless of extraction method used, and a correlation of 0.92 (p < 0.0001) is seen on comparison of fresh-frozen tissues extracted with different methods. A comparison of the summary statistics using mean Ct values obtained for each extraction method between tumour and control samples was also performed (Figure 3).

**Figure 4:**
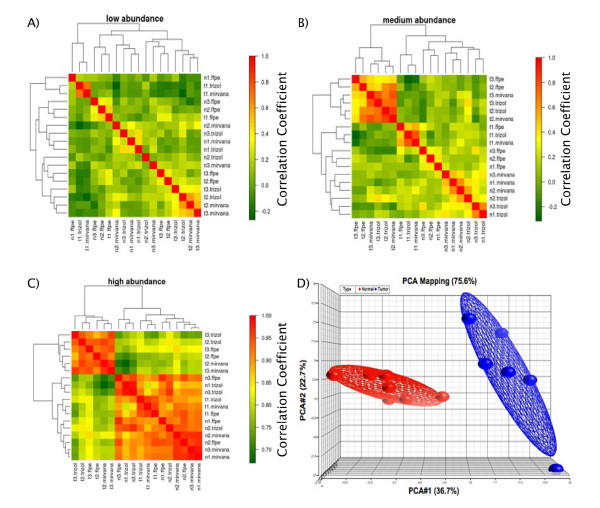
**Correlation heatmaps and PCA mapping for paired fresh-frozen and FFPE tissues**. Correlation heat-maps demonstrating hierarchical clustering among 3 normal lymph nodes (n1-n3) and 3 mantle cell lymphomas (t1-t3) extracted using different techniques using: A) Low abundance miRs B) Medium abundance miRs C) High abundance miRs. Panel D) shows principal component analysis (PCA) confirming the similarity of miR expression between tumours (red), and normal samples (blue), irrespective of tissue origin (FFPE vs. fresh-frozen).

Unsupervised hierarchical clustering analysis was performed (*hclust *R function, one minus Pearson correlation distance measure) for tumours and normal samples, taking into account the extraction method used. Figures 4A-C are heatmaps showing the results for the low, medium and high abundance miRs. Principal component analysis (PCA) was also applied, and confirmed that all tumours group together and separate from normal samples, independent of the tissue specimen and method used for RNA extraction (Figure 4D).

**Figure 5:**
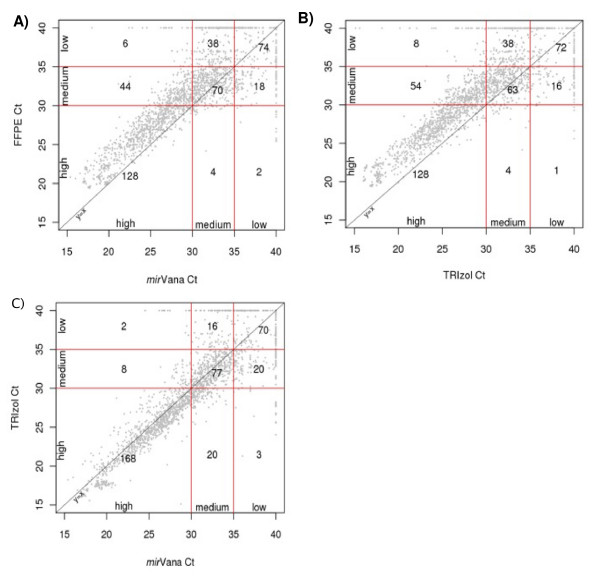
**Comparison of miR abundance in paired fresh-frozen and FFPE tissues, using different RNA extraction methods**. Plots show the comparison of Ct values for three normal and three mantle cell lymphoma samples by the three different extraction methods: A) FFPE (RecoverAll) vs. mirVana; B) FFPE (RecoverAll) vs. TRIzol; C) TRIzol vs. mirVana. Red lines delineate the abundance strata for each extraction method, with numbers indicating the average number of miRs per sample in each zone. For example, in the FFPE vs. mirVana plot (Figure 5A), 44 miRs are high abundance by mirVana and medium abundance by FFPE (RecoverAll), but only 4 miRs are high abundance by FFPE (RecoverAll) and medium abundance by mirVana.

Finally, the number of detectable miRs in each abundance stratum was determined for FFPE compared to fresh-frozen samples, using the results from all 3 normal benign lymph nodes and all 3 mantle cell lymphoma samples (Table 1 and Figure 5A-C). From this analysis, we detected a similar number of medium abundance miRs regardless of tissue source and extraction method used. Similar numbers of miRs are seen in all abundance strata among fresh-frozen samples regardless of extraction method used, whereas FFPE samples tend to have an increase in the number of low abundance miRs and a decreased number of high abundance miRs. Chi-square analysis demonstrates that the shift in proportion of miRs in the three strata is significantly different between fresh-frozen and FFPE tissues [TRIzol and FFPE (χ^2 ^= 17.5, df = 2, p = 0.0002); *mir*Vana and FFPE (χ^2 ^= 13.5, df = 2, p = 0.001)], but not between TRIzol and *mir*Vana (χ^2 ^= 0.32, df = 2, p = 0.85). This difference may be due to reduced RNA yields in FFPE samples, which is known to occur and has been reported in the literature [7-10].

**Table 1:** Numbers of miRs in each abundance stratum according to different extraction methods used.

	Extraction method		
	**RecoverAll protocol** **(FFPE)**	***mir *Vana protocol** **(Fresh-frozen tissue)**	**TRIzol-Qiagen** **(Fresh-frozen tissue)**

Low	125	96	90

Medium	141	122	122

High	118	166	172

## Discussion

Recent studies have shown that miR expression profiling may be more accurate for distinguishing disease states than mRNA expression analysis [23,24]. Since miRs are better preserved, and can be obtained from FFPE tissue, they may be a better choice for expression studies when using FFPE samples [11]. miR expression has been examined in FFPE and fresh-frozen tissue specimens. For example, an 89% correlation in miR expression profiles was shown between FFPE and fresh-frozen samples from murine liver, using a locked nucleic acid (LNA)-based miR microarray platform [11]. Other studies, using human tissue sources (colon, myometrium, lymph node, melanocytes), on various microarray platforms (*mir*Vana miRNA Bioarrays, Agilent human miRNA arrays, and Invitrogen Ncode Multi-Species miRNA microarrays), showed high correlation between miR expression profiles in FFPE and fresh-frozen samples [18-20]. Technologies such as Luminex's fluorescence labeled beads, when used on FFPE and fresh-frozen breast tissue, also yielded similar results [15], showing that FFPE tissues are useful for miR expression analysis.

Another platform for miR expression analysis, using high-throughput qRT-PCR, has yielded high correlation coefficients when comparing fresh-frozen and FFPE samples [12,16,17]. Recently, a comparison between FFPE and fresh-frozen tissue using two pairs of breast carcinoma samples was performed on the high-throughput qRT-PCR TaqMan Human MicroRNA Array v1.0 (Early Access), demonstrating a correlation of 0.94 between paired samples [21]. In our study, we also tested the TaqMan Human MicroRNA Array v1.0 (Early Access) platform to show its utility and reproducibility in determining a miR expression profile using FFPE clinical samples. However, our study differs in several aspects, including the assessment of different extraction methods and demonstration of platform optimization using less than recommended RNA amounts as described in Materials and Methods. These experiments are necessary to warrant the use of this platform in the study of miR expression in FFPE tissues, and have not been previously described. Our study also adds new information to the current literature, showing the importance of stratifying miRs based on abundance when analyzing miR expression data.

Our optimization results showed that the amount of input RNA and dilution of the cDNA can affect miR expression levels detected. These effects are more pronounced if examined from the perspective of miR abundance. Our results show that input RNA from FFPE samples should be maximized and that dilution of cDNA should be kept to a minimum, in order to generate the most reliable and reproducible miR expression profiles using the TaqMan Human MicroRNA Array v1.0 (Early Access) platform. This is apparent when observing the shifts that occur in miR abundance with changes in RNA input concentration. As seen in Figure 1A, with a lower input RNA concentration, a lower number of high abundance miRs are detected. Our results showed a significant shift in the proportion of miRs in each abundance stratum with the increase in input RNA concentration from 10 ng/μL to 200 ng/μL (chi-square test, χ^2 ^= 12.7, df = 2, p = 0.002). The largest change is decreasing frequency of low abundance miRs (163 to 130) and increasing frequency of high abundance miRs (72 to 112), a pattern which is consistent over each increment of input RNA concentration. Of note, the low abundance strata across the input RNA concentrations have a higher variability, compared to the medium and high abundance strata at the same concentrations, as shown in Figure 1B. This demonstrates that expression values obtained for miRs within the medium and high abundance strata are more reliable for analysis and a direct link between increasing input RNA concentration and improved reliability for low and medium abundance miRs.

Well failures are also less common as input RNA concentrations increase or cDNA dilutions decrease. Interestingly, dilution of cDNA can be used in order to compensate for low input RNA amounts, a scenario which can occur should RNA yield be low. This can be observed when comparing an input RNA concentration of 200 ng/μL with a 15× cDNA dilution factor and an input RNA concentration of 66.7 ng/μL with a 5× cDNA dilution factor. Our results show the consistency of Ct values obtained with different cDNA dilution factors (Pearson correlation 0.95). This was also shown to be true when examining correlation coefficients relative to miR abundance. There was also no statistically significant shift in the proportion of miRs in each abundance stratum between equivalent combinations of input RNA concentration and cDNA dilution [chi-square test, (χ^2 ^= *3.3, df = 6, p = 0.77*)]. In our laboratory, we have optimized the experiments to use an input RNA concentration of 200 ng/μL with a 15× cDNA dilution factor. This gives us the flexibility to use input RNA concentrations as low as 60 ng/μL with a 5× cDNA dilution factor, which is the lowest cDNA dilution possible according to the manufacturer's protocol, in order to maintain equivalent miR profiles between samples.

Finally, we showed that miR expression was highly correlated in FFPE and fresh-frozen lymphoid tissues, regardless of the extraction method used, despite the fact that raw Ct scores obtained from FFPE samples are significantly different than those from fresh-frozen tissue. This is likely due to reduced yields of RNA from FFPE tissue. Unsupervised hierarchical clustering analysis, using correlation heat-maps, showed that tumour and normal samples clustered in separate groups. The hierarchical clustering performed according to miR abundance demonstrated that the medium abundance miRs appear to be most informative in helping to segregate tumours from normal samples. This may be because many miRs that fall into the low abundance category are potentially not expressed in lymphoid tissue. Our pre-filtering removed miRs with Ct values of 40 in all twelve samples, meaning that if one miR had a Ct value less than 40 but greater than 35 in even one sample, it would be included in the analysis as a low abundance miR. The high abundance miRs may be informative as well, although one tumour sample clusters together with the normal samples. Interestingly, this tumour is a post-treatment sample; the patient having been treated with R-CHOP (rituximab-cyclophosphamide-doxorubicin-vincristine-prednisone) prior to biopsy and sample collection. Rituximab is an anti-CD-20 antibody, which targets B-lymphocytes expressing CD20 (including mantle cell lymphoma), and combined with CHOP chemotherapy, it has been shown to improve overall and complete response rates, as well as time to treatment failure, in mantle cell lymphoma patients [25,26].

Also of note is the shift of miRs from high to medium and medium to low abundance in FFPE compared to fresh-frozen tissue, seen in Table 1 and Figure 4. The shift in proportion of miRs in the three strata is significantly different by chi-square test between TRIzol and FFPE (χ^2 ^= 17.5, df = 2, p = 0.0002) and between *mir*Vana and FFPE (χ^2 ^= 13.5, df = 2, p = 0.001), but not between TRIzol and *mir*Vana (χ^2 ^= 0.32, df = 2, p = 0.85). This decrease in the proportion of high abundance miRs and increase in the proportion of low abundance miRs may be due to known reduction of RNA yields that occur in FFPE tissue [7-10]. Reduction in RNA yield is seen due to chemical modification of RNA introduced by formalin fixation. This modification occurs through the addition of mono-methylol (-CH_2_OH) groups to RNA, as well as formation of secondary modifications such as methylene bridging, preferentially at adenine residues [8]. Although miRs may be relatively protected due to association with other proteins (e.g. the RISC complex), some modifications likely occur since the shift in miR abundance from high to medium and from medium to low indicates a reduction in miR yield from FFPE samples. This effect has also been seen by Hoefig et al. [16] who demonstrated a 1.0-1.5 Ct reduction in miR expression between fresh-frozen tissues and their paired FFPE samples. In our study we observe that this reduction does not affect sample biology. Our PCA results (Fig. 4D) reveal a complete separation between normals and tumours regardless of sample treatment (FFPE *vs*. fresh-frozen). Similar to our results, Hoefig et al. [16] demonstrated that biologically relevant miR expression profiles between liver and lymph node tissues were unchanged regardless of tissue treatment, and that this biological variance was higher than the technical variance introduced by formalin fixation and paraffin embedding [16]. In our study, by comparing normal and tumoural tissues from the same source (lymphoid), we were able to demonstrate robust differential expression, which was higher than the differences between FFPE and fresh-frozen tissue, as seen in Figure 4. Thus, the correlations reported in our study demonstrate that miR levels likely reflect the disease state in patients regardless of tissue source (FFPE *vs*. fresh-frozen) used for analysis.

Although our results show that the technical differences introduced by tissue treatment are minimal, we also show that data analysis can be strengthened by examining miR expression according to abundance strata. As seen in Figure 4, our results indicate that stratification of miRs according to abundance should be considered during data analysis as low abundance miRs (Ct>35) do reveal low correlation coefficients when compared to medium (30≤Ct≤35) and high abundance miRs (Ct<30). Based on our results, we would exclude the low abundance miRs from analysis as the miRs in the medium and high abundance strata show superior correlations and sample clustering. We suggest that investigators stratify their miR expression data and judge whether or not to include miRs from all strata during data analysis on a case-by-case basis. This method of data analysis suggested by us is a potential tool for further optimization of miR expression data derived from FFPE samples and will increase the correlation between these samples and their paired fresh-frozen tissues.

## Conclusions

Overall, our study demonstrates the utility of the high-throughput, qRT-PCR-based platform for miR expression using FFPE clinical samples. The expression profiles obtained from the arrays are reproducible, demonstrating high correlation values both within the same samples and between samples. Expression profiles generated through this high-throughput method can be used to discern between different disease states (benign and malignant). Of note, RNA input concentrations used on this platform should be maximized whenever possible, and cDNA dilution factors should be kept to a minimum.

Given the availability of FFPE samples in laboratories worldwide, and the amenability of array platforms for the use of FFPE samples, we believe that optimization and use of high-throughput analysis, such as miR expression will facilitate further insight into disease biology.

## Methods

### Samples

Samples were collected at surgical resection and ranged from 1 to 8 years in age. Samples were divided into two specimens at collection: one used for formalin fixation and paraffin embedding, the other placed in Optimal Cutting Temperature (OCT) compound and stored in liquid nitrogen until RNA extraction. FFPE samples from four benign, reactive lymph nodes and three mantle cell lymphomas were obtained from the Department of Pathology at the University Health Network with the approval of the Research Ethics Board. Three to five 10 μm sections were obtained and kept under RNase-free conditions until RNA extraction. Paired fresh-frozen and FFPE samples (3 benign reactive lymph nodes and 3 mantle cell lymphomas) were used for comparison. Mantle cell lymphoma samples contained >80% tumour cells in all sections. One case (t1) was a post-treatment sample. In addition, Universal RNA (Stratagene), which is a pooled RNA from 10 cancer cell lines, was used as a positive amplification control.

### RNA extraction

RNA from FFPE samples was extracted using the RecoverAll Total Nucleic Acid Isolation Kit (Ambion), as it has been shown to be the preferred RNA extraction method for FFPE samples [7]. Modifications to the manufacturers' protocol were carried out as follows in order to improve RNA yield: 1 mL of xylene was added to each sample and the mixture heated at 50°C for 5 minutes to melt the paraffin. The samples were centrifuged at maximum speed twice for 2 minutes each to pellet the sample. The xylene was removed and samples were washed twice in 100% ethanol with centrifugation at 12,000 rpm. The pellet was dried at 37°C for 10-15 minutes prior to addition of 400 μL of Digestion Buffer and 8 μL of Protease provided in the kit. The samples were then incubated at 50°C overnight in order to obtain complete digestion of the samples. The remainder of the protocol was carried out as per manufacturer's instructions with the exception of the elution step in which the samples were eluted in a total of 30 μL RNase-free water (Sigma) instead of the recommended 60 μL.

RNA from the fresh-frozen samples was obtained using two different extraction methods. We wished to examine whether or not our in-house RNA extraction method (TRIzol followed by Qiagen purification) would yield similar results to the commercially available *mir*Vana Total RNA Isolation kit as used by Doleshal et al. 2008. Samples were ground with the use of liquid nitrogen and a mortar and pestle. Half of each sample was placed in either 300 μL of Lysis Buffer (*mir*Vana Total RNA Isolation Procedure protocol; Ambion) or 1 mL TRIzol (Invitrogen). The samples were then homogenized using a mortar and pestle. RNA extraction using the *mir*Vana Total RNA Isolation Procedure was performed as per manufacturer's instructions. The TRIzol extraction protocol was carried out by incubating the sample in TRIzol reagent at RT for 5 minutes, followed by addition of 200 μL of chloroform to, the mixture, which was shaken vigorously for 15 seconds, incubated at RT for 3 minutes, and centrifuged at 12,000 × g at 4°C for 15 minutes. The upper aqueous phase was then removed, transferred to a fresh tube, and 500 μL of isopropyl alcohol was added. The mixture was then incubated at -20°C for 15 minutes prior to placing it on an RNeasy spin column (Qiagen). The remainder of the protocol was carried out using the instructions provided with the RNeasy Mini kit (Qiagen), with the exception of the elution step, which used a total volume of 40 μL RNase-free water (Sigma). The concentration and purity of all RNA samples were assessed using the NanoDrop spectrophotometer (NanoDrop Technologies Inc.).

### Generation of miR expression data using the TaqMan Human MicroRNA Array

Generation of miR expression data was a two-step process involving a reverse transcription reaction followed by a quantitative real-time polymerase chain reaction (qRT-PCR). The reverse transcription reaction was carried out according to the manufacturer's instructions including 1 mM dNTPs (including TTP), 50 U MultiScribe Reverse Transcriptase, 3.8 U RNase inhibitor, and 1× Reverse Transcription buffer. The manufacturer suggests the use of 10-100 ng/μL of input RNA for each reverse transcription reaction. TaqMan stem-loop primers for 365 individual miRs and 3 endogenous controls were used in the reverse transcription reaction. Conditions for the reverse transcription reaction were as follows: 16°C for 30 minutes, 42°C for 30 minutes, 85°C for 5 minutes, then hold at 4°C. cDNA samples were stored at -20°C overnight prior to application on the TaqMan Human MicroRNA Array v1.0 (Early Access). The TaqMan Human MicroRNA Array v1.0 (Early Access) [TaqMan array] is a 384-well microfluidics card containing 365 primer-probe sets for individual miRs as well as duplicates of 3 primer-probe sets for endogenous small nucleolar RNAs and duplicate negative control blank wells. Reverse transcription reaction products were diluted (62.5×, 30×, 15×, or 5×) prior to adding an equivalent volume of TaqMan 2× Universal PCR Master Mix, No AmpErase UNG. A minimum of 5× cDNA dilution is required to obtain the necessary volume for application to the TaqMan Human MicroRNA Array. The entire mixture was then added to individual ports of the TaqMan Human MicroRNA Array v1.0 (Early Access); the array was centrifuged at 1,200 rpm twice for 1 minute each, and then run on an Applied Biosystems 7900HT Fast Real-Time PCR system for 40 cycles. Raw microRNA data (GEO series record GSE22264) are available for download at http://www.ncbi.nlm.nih.gov/geo/.

### Statistical and bioinformatics analysis

Mixed model regression analysis was used to adjust for repeated measures of the same sample and the potential dilution effects. This, as well as bar plots, were generated using SAS software, Version 9.1 of the SAS System, Copyright ^©^2009 SAS Institute Inc. SAS and all other SAS Institute Inc. product or service names are registered trademarks or trademarks of SAS Institute Inc., Cary, NC, USA [27]. 3-D principal component analysis (PCA) was performed using Partek^® ^software, version 6.4 Copyright ^© ^2009 Partek Inc., St. Louis, MO, USA [28].

All other statistical analysis and plotting were performed using the R statistical environment (R Development Core Team, 2008, v2.8.1) [29], on the IBM HS21XM BladeCentre cluster running CentOS 5.1. Additional plotting functions were used from the fields (v.5.0.2) [30] and RColorBrewer (v1.0-2) [31], R packages.

## Competing interests

The authors declare that they have no competing interests.

## Authors' contributions

RSG designed the study, helped collect and extract samples, ran the TLDA assays, helped with data analysis and drafted the manuscript. LW performed the data analysis and helped draft the manuscript. JM and NKC helped with sample extraction and data analysis. WX performed statistical analysis and helped draft the manuscript. PPR helped with study design, data interpretation and helped draft the manuscript. DJB contributed with sample collection and pathological diagnosis. IJ and MRC helped coordinate the study. SKR helped design and supervised the study and gave final approval of the manuscript. All authors read and approved the manuscript.

## Supplementary Material

Additional file 1 **Figure S1**. Absolute value of the difference between duplicate measurements as a function of the duplicate mean for all input RNA concentrations. Measurements where at least one of the duplicate Ct values equals 40 are removed. The red line shows a cubic spline fit using default settings for the smooth spline function in the R statistics package (R Development Core Team, 2008). The apparent decrease in variability for mean Ct>37 is an artifact caused by the absence of Ct values greater or equal to 40. Click here for file

Additional file 2 **Figure S2**. A) Graph of average percentage of well failures according to input RNA concentrations. Note that cDNA dilution factors were kept constant (15×). B) Percentage of well failures as they relate to input RNA concentration and cDNA dilution factor. cDNA dilution factors are shown on the x-axis and percentage of well failures are shown on the y-axis. Click here for file

Additional file 3 **Table S1**. Table of Pearson correlation coefficients between equivalent input RNA concentration/cDNA dilution factor combinations (equivalent samples). Click here for file

Additional file 4 **Table S2**. Table depicting number of miRs in each abundance stratum for equivalent dilutions. No significant difference exists in the abundance distributions of the columns by chi-square analysis (χ^2 ^= 3.3, df = 6, p = 0.77). Click here for file
