# Supplementary figures and images for: Optimization and analysis of a quantitative real-time PCR-based technique to determine microRNA expression in formalin-fixed paraffin-embedded samples
Source: BMC Biotechnol. 2010 Jun 23;10:47. doi: 10.1186/1472-6750-10-47 (PMC2902407; doi:10.1186/1472-6750-10-47)

## Slide 1
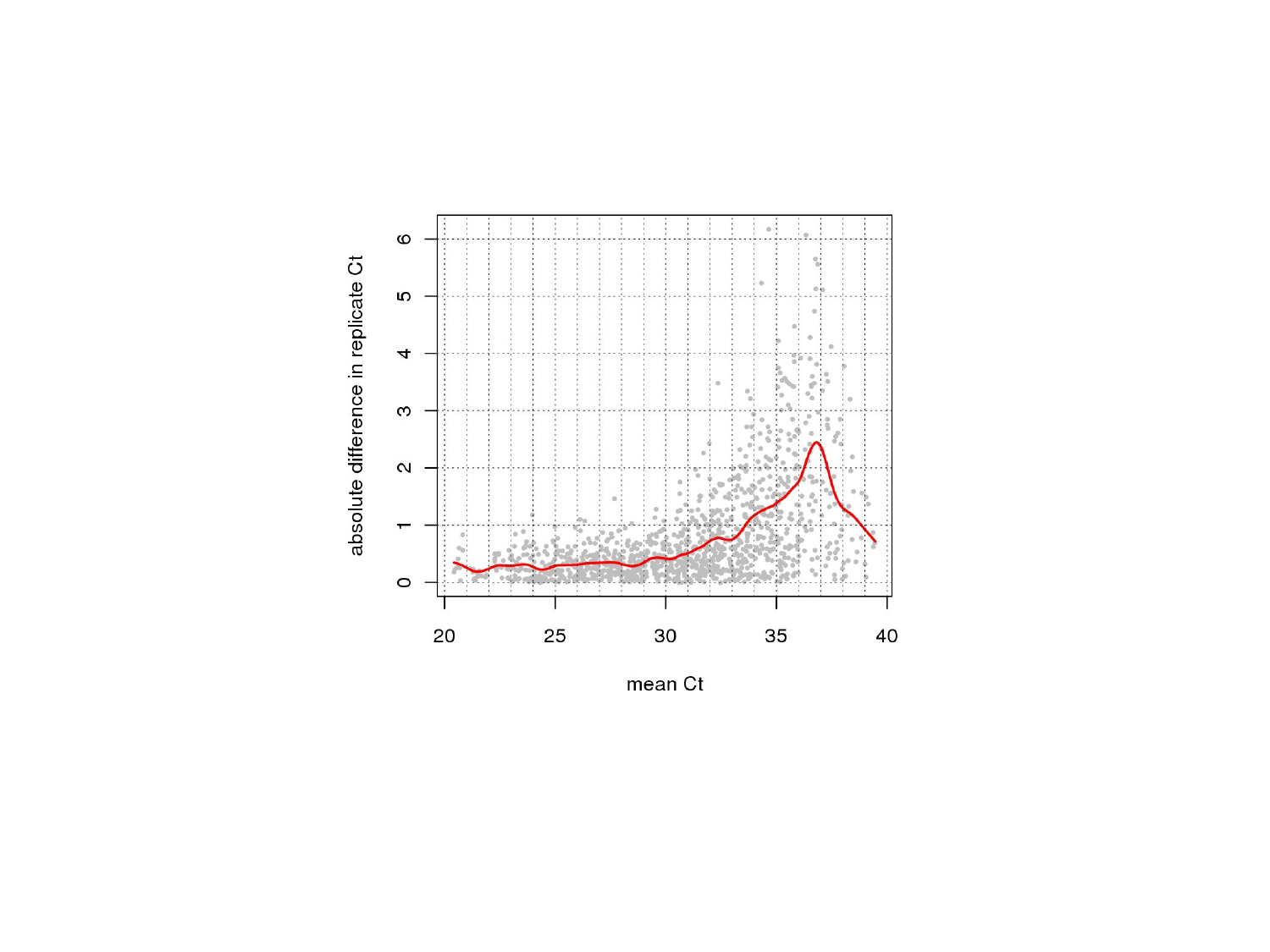

Supplement: Additional file 1 — Figure S1. Absolute value of the difference between duplicate measurements as a function of the duplicate mean for all input RNA concentrations. Measurements where at least one of the duplicate Ct values equals 40 are removed. The red line shows a cubic spline fit using default settings for the smooth spline function in the R statistics package (R Development Core Team, 2008). The apparent decrease in variability for mean Ct>37 is an artifact caused by the absence of Ct values greater or equal to 40. [file 1472-6750-10-47-S1.PPT]

## Slide 1
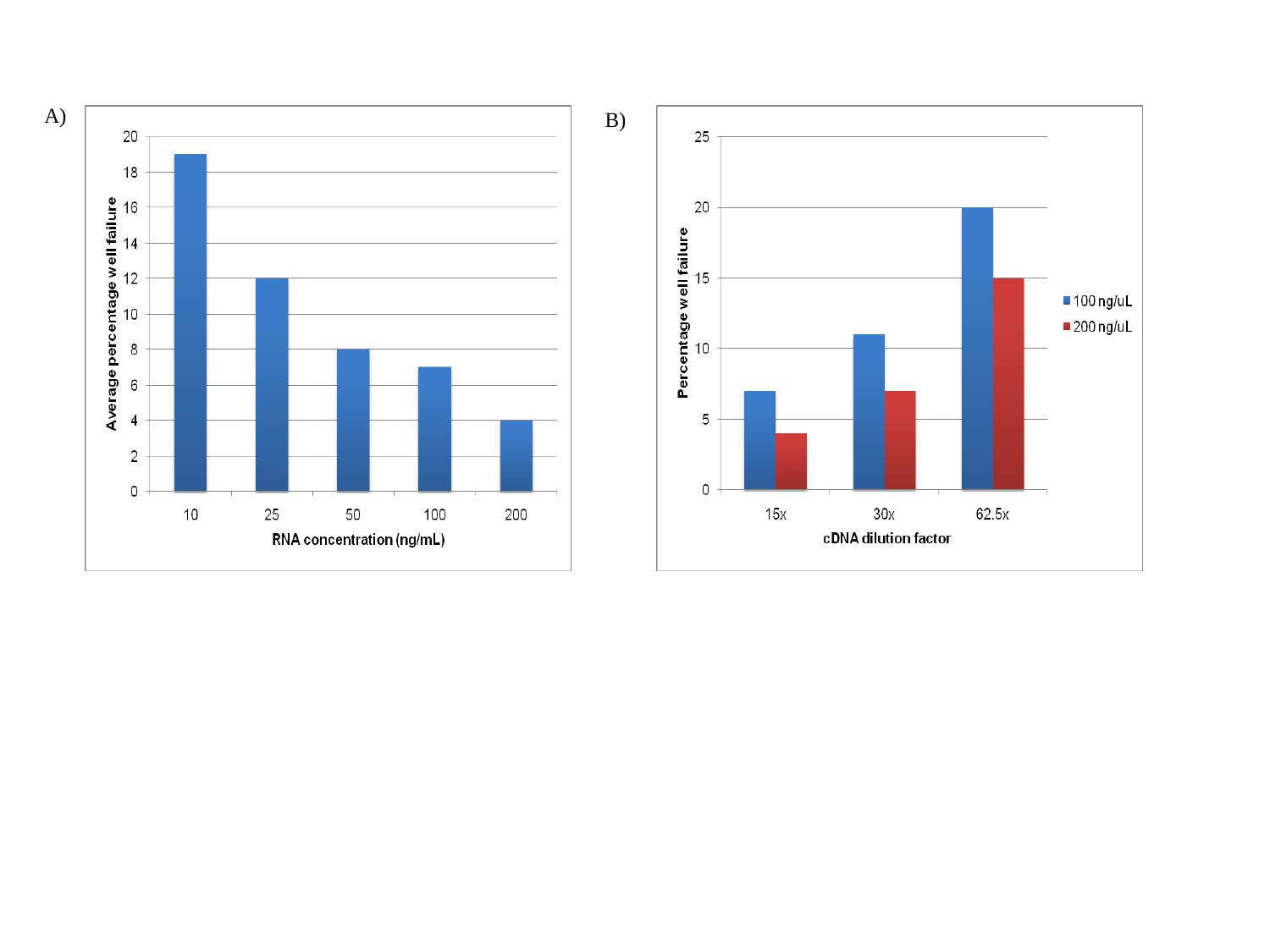

A)
B)

Supplement: Additional file 2 — Figure S2. A) Graph of average percentage of well failures according to input RNA concentrations. Note that cDNA dilution factors were kept constant (15×). B) Percentage of well failures as they relate to input RNA concentration and cDNA dilution factor. cDNA dilution factors are shown on the x-axis and percentage of well failures are shown on the y-axis. [file 1472-6750-10-47-S2.PPT]
