# Supplementary material for: Optimization and analysis of a quantitative real-time PCR-based technique to determine microRNA expression in formalin-fixed paraffin-embedded samples
Source: BMC Biotechnol. 2010 Jun 23;10:47. doi: 10.1186/1472-6750-10-47 (PMC2902407; doi:10.1186/1472-6750-10-47)
Supplement: Additional file 3 — Table S1. Table of Pearson correlation coefficients between equivalent input RNA concentration/cDNA dilution factor combinations (equivalent samples). [file 1472-6750-10-47-S3.DOC]

*Table S1. Pearson correlation coefficients between equivalent input RNA concentration/cDNA dilution factor combinations (equivalent samples).*

|  |  | RNA concentration (ng/μL)/cDNA dilution factor | |
| --- | --- | --- | --- |
|  |  | 100/7.5x | 66.7/5x |
| RNA concentration (ng/μL)/cDNA dilution factor | 200/15x | 0.96 | 0.95 |
|  | 100/7.5x | 1 | 0.944 |
