# Supplementary material for: Optimization and analysis of a quantitative real-time PCR-based technique to determine microRNA expression in formalin-fixed paraffin-embedded samples
Source: BMC Biotechnol. 2010 Jun 23;10:47. doi: 10.1186/1472-6750-10-47 (PMC2902407; doi:10.1186/1472-6750-10-47)
Supplement: Additional file 4 — Table S2. Table depicting number of miRs in each abundance stratum for equivalent dilutions. No significant difference exists in the abundance distributions of the columns by chi-square analysis (χ2 = 3.3, df = 6, p = 0.77). [file 1472-6750-10-47-S4.DOC]

*Table S2. Number of miRs in each abundance stratum for equivalent dilutions. No significant difference exists in the abundance distributions of the columns by chi-squared analysis (χ^2^=3.3, df=6, p=0.77).*

|  | Sample (RNA concentration (ng/μL)/cDNA dilution factor) | | | |
| --- | --- | --- | --- | --- |
|  | 200/15x (1) | 200/15x(2) | 100/7.5x | 66.7/5x |
| Low | 179 | 184 | 170 | 163 |
| Medium | 93 | 89 | 98 | 106 |
| High | 112 | 111 | 116 | 115 |
